# Effect of polymorphism in *Rhinolophus affinis* ACE2 on entry of SARS-CoV-2 related bat coronaviruses

**DOI:** 10.1371/journal.ppat.1011116

**Published:** 2023-01-23

**Authors:** Pei Li, Jiaxin Hu, Yan Liu, Xiuyuan Ou, Zhixia Mu, Xing Lu, Fuwen Zan, Mengmeng Cao, Lin Tan, Siwen Dong, Yao Zhou, Jian Lu, Qi Jin, Jianwei Wang, Zhiqiang Wu, Yingtao Zhang, Zhaohui Qian

**Affiliations:** 1 NHC Key Laboratory of Systems Biology of Pathogens, Institute of Pathogen Biology, Chinese Academy of Medical Sciences & Peking Union Medical College, Beijing, China; 2 College of Life Sciences, Peking University, Beijing, China; 3 School of Pharmaceutical Sciences, Peking University, Beijing, China; University of Texas Medical Branch at Galveston, UNITED STATES

## Abstract

Bat coronavirus RaTG13 shares about 96.2% nucleotide sequence identity with that of SARS-CoV-2 and uses human and *Rhinolophus affinis* (Ra) angiotensin-converting enzyme 2 (ACE2) as entry receptors. Whether there are bat species other than *R*. *affinis* susceptible to RaTG13 infection remains elusive. Here, we show that, among 18 different bat ACE2s tested, only RaACE2 is highly susceptible to transduction by RaTG13 S pseudovirions, indicating that the bat species harboring RaTG13 might be very limited. RaACE2 has seven polymorphic variants, RA-01 to RA-07, and they show different susceptibilities to RaTG13 S pseudovirions transduction. Sequence and mutagenesis analyses reveal that residues 34, 38, and 83 in RaACE2 might play critical roles in interaction with the RaTG13 S protein. Of note, RaACE2 polymorphisms have minimal effect on S proteins of SARS-CoV-2 and several SARS-CoV-2 related CoVs (SC2r-CoVs) including BANAL-20-52 and BANAL-20-236 in terms of binding, membrane fusion, and pseudovirus entry. Further mutagenesis analyses identify residues 501 and 505 in S proteins critical for the recognition of different RaACE2 variants and pangolin ACE2 (pACE2), indicating that RaTG13 might have not been well adapted to *R*. *affinis* bats. While single D501N and H505Y changes in RaTG13 S protein significantly enhance the infectivity and minimize the difference in susceptibility among different RaACE2 variants, an N501D substitution in SARS-CoV-2 S protein displays marked disparity in transduction efficiencies among RaACE2 variants with a significant reduction in infectivity on several RaACE2 variants. Finally, a T372A substitution in RaTG13 S protein not only significantly increases infectivity on all RaACE2 variants, but also markedly enhances entry on several bat ACE2s including *R*. *sinicus* YN, *R*. *pearsonii*, and *R*. *ferrumeiqunum*. However, the T372A mutant is about 4-fold more sensitive to neutralizing sera from mice immunized with BANAL-20-52 S, suggesting that the better immune evasion ability of T372 over A372 might contribute to the natural selective advantage of T372 over A372 among bat CoVs. Together, our study aids a better understanding of coronavirus entry, vaccine design, and evolution.

## Author summary

Bat CoV RaTG13 has zoonotic transmission potential, and interactions between S protein and ACE2 play essential roles. RaTG13 uses RaACE2 as an entry receptor, and there are at least seven polymorphic RaACE2 variants. The effect of RaACE2 polymorphism on entry of RaTG13 and other SARS-CoV-2 related bat CoVs (SC2r-CoVs) has not been determined. In this study, we find that RaTG13 S pseudovirions only highly efficiently transduce cells expressing RaACE2 among available 18 bat species commonly habituating in Southwestern China and Southeastern Asia, suggesting that there might be limited bat species including *R*. *affinis* susceptible to infection of RaTG13 virus. The RaACE2 polymorphism has a marked effect on the entry of RaTG13, whereas it shows a minimal effect on the entry of SARS-CoV-2 and SC2r-CoVs like BANAL-20-52, BANAL-20-236, and pangolin-CoV GD. Further sequence and mutagenesis analyses identify residues 34, 38, and 83 in RaACE2 and residues 501 and 505 in the S proteins critical for S protein and receptor interactions, indicating that RaTG13 might have not been well adapted to *R*. *affinis* bats. We also find that T372A substitution in RaTG13 S significantly enhances entry on all RaACE2 variants and several bat ACE2s, and immune evasion might contribute to the natural selection of T372 over A372 in bat S proteins. Altogether, our work provides detailed analyses of the effect of RaACE2 polymorphism on the entry of RaTG13 and other SC2r-CoVs and a better understanding of the entry and evolution of CoVs.

## Introduction

Coronaviruses (CoVs) are positive-sense single stranded RNA viruses and currently classified into four genera, alpha-coronavirus (α-CoV), beta-coronavirus (β-CoV), gamma-coronavirus (γ-CoV), and delta-coronavirus (δ-CoV) [1].The α-CoV and β-CoV only infect mammals, whereas γ-CoV and δ-CoV mainly infect birds [2]. Some δ-CoVs, like porcine δ-CoV, also infect mammals [3,4]. Since the beginning of the 21st century, there have been three outbreaks caused by β-CoVs, severe acute respiratory syndrome coronavirus (SARS-CoV) in 2003–2004 [5–7], Middle East respiratory syndrome coronavirus (MERS-CoV) since 2012 [8], and severe acute respiratory syndrome coronavirus 2 (SARS-CoV-2) since the end of 2019 [9–11]. All three CoVs likely originated from bats and might spread to a human through an intermediate host(s) [12]. Bat CoV RaTG13 was found in a specimen from *Rhinolophus affinis* bat in China’s Yunnan province and its genome shares 96.2% of nucleotide sequence identity with that of SARS-CoV-2, indicating likely bat origin of SARS-CoV-2 [11,13,14].

Receptor interaction is the first and essential step for viral infection. Both SARS-CoV-2 and RaTG13 can use human angiotensin-converting enzyme 2 (hACE2) as the entry receptor [11,15–17]. Recently we showed that *Rhinolophus affinis* ACE2 (RaACE2) can also serve as the functional entry receptor for SARS-CoV-2 and RaTG13 [18]. Although the function and sequences of ACE2s are highly conserved among different mammalian species, polymorphism is also a common phenomenon present in ACE2 of individual species. The ACE2 polymorphisms have been detected in several bat species, such as *R*.*sinicus*, *R*.*ferrumequinum*, *R*.*affinis*, *Hipposideros armiger*, and so on [19, 20]. There are at least seven RaACE2 variants, and their roles in bat coronavirus RaTG13 entry have not been studied. Here, we determined their effect on receptor binding, cell-cell fusion, and pseudovirion entry of SARS-CoV-2 and RaTG13 S protein, identified residues 34, 38, and 83 in RaACE2 and residues 501 and 505 in the S proteins critical for S protein and receptor interaction, and found that immune evasion might contribute to evolutionary advantage of T372 over A372 in RaTG13 S protein.

## Results

### Evaluation of susceptibility of 18 bat ACE2s to RaTG13 infection

**Fig 1:**
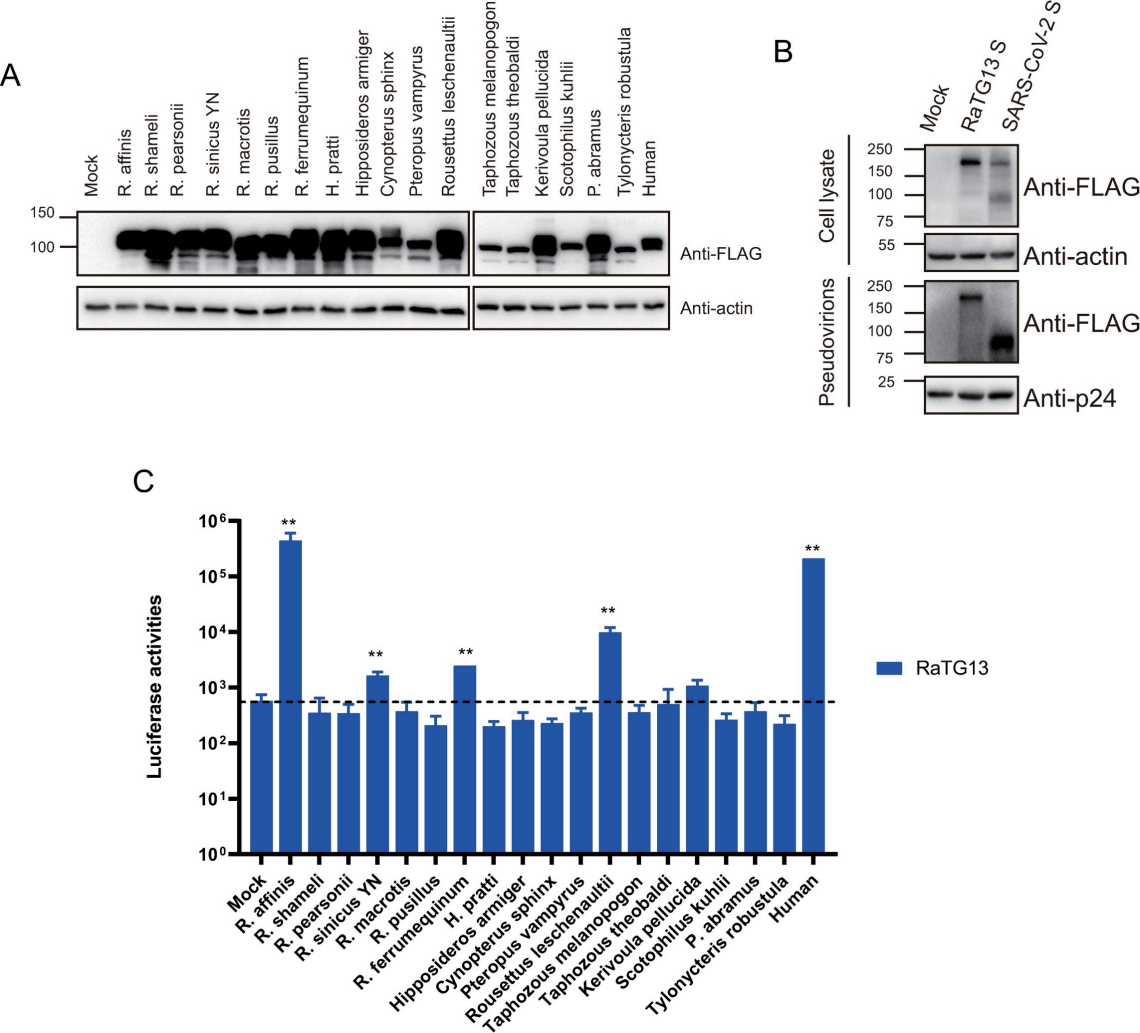
Expressions and transduction of bat ACE2s by RaTG13 S pseudovirons. (A) Western blot analysis of expression of different bat ACE2s in HEK293 cells. HEK293 cells were transfected with plasmids encoding FLAG-tagged bat ACE2s by PEI and lysed at 40 hrs post-transfection. Proteins were detected using anti-FLAG M2 antibody. The β-actin was used as a loading control. (B) Detection of the S proteins of RaTG13 and SARS-CoV-2 in celllysates and pseudovirions by western blot. HEK293T cells transfected with either empty vector or plasmids encoding the indicated CoV S proteins were harvested at 40 h post-transfection. The S proteins in cell lysate and pseudovirions were subjected to western blot analysis using an anti-FLAG M2 antibody. β-Actin and gag-p24 served as loading controls. The full-length S protein is about 180 kD, while the cleaved S protein is about 90 kD. Experiments were done three times and the representative was shown. (C) Entry mediated by the S protein of RaTG13 into cells expressing different bat and human ACE2s. HEK-293 cells transiently expressing bat and human ACE2s were transduced with RaTG13 S pseudovirions and the transduction efficiency was detected 40 hrs later and manifested as luciferase activities. Experiments were done in triplicate and repeated at least three times. One representative is shown with error bars indicating SEM. Statistical significance is set as * p<0.05 and ** p<0.01 and calculated by T-test.

Although RaTG13 was first identified in *Rhinolophus affinis* bat, could any other bat species be susceptible to RaTG13 infection? We obtained ACE2 sequences of 18 different bat species available in GenBank (Table 1), which also commonly inhabit southwestern China and Southeast Asia [21]. The plasmids encoding each bat ACE2s were transfected into 293 cells and their susceptibilities were evaluated using RaTG13 S pseudovirions. The majority of bat ACE2s were expressed at levels similar to or better than hACE2, although the level of ACE2s from *T*. *melanopogon*, *T*. *theobaldi*, *S*. *kuhlii*, *T*. *robustula* was marked lower than hACE2 (Fig 1A). Both SARS-CoV-2 and RaTG13 S proteins were incorporated into pseudovirions well (Fig 1B). Consistent with our previous report [18], RaTG13 S pseudovirions used hACE2 and RaACE (RA-07) for virus entry efficiently and their luciferase activities were increased by 368 and 770-fold (Fig 1C), respectively, compared to mock controls. To our surprise, besides RaACE2, only *Rousettus leschenaultia*, a fruit bat, ACE2 showed a more than 10-fold increase in luciferase activity, ACE2s from *R*. *sinicus YN*, *R*. *ferrumequinum*, and *Kerivoula pellucida* showed 2.9, 4.3, and 1.9-fold increase in transduction over control, respectively, and the remaining 13 bat ACEs only gave background level of infection (Fig 1C). There are at least 8 variants of *R*. *sinicus YN* ACE2 [22]. Besides *R*. *sinicus YN* ACE2 (RS-allele7), we further characterized two additional *R*. *sinicus* ACE2 variants (RS-allele4 and RS-allele8) [22], and found that both only showed background level of susceptibility (S1 Fig). The total frequency of three RS-alleles accounts for about 52% of *R*. *sinicus* population (28% for allele4, 8% for allele7, and 16% for allele8), and the poor transduction of all three variants by RaTG13 S pseudoviruses indicates that *R*. *sinicus* ACE2s might not be highly susceptible to RaTG13 infection. Together, these results indicate that very limited bat species might be susceptible to infection of RaTG13 virus and *R*. *affinis* bat might be one of bat species harboring RaTG13 virus.

**Table 1:** Accession number for 18 different bat species.

Bat species	Accession
*Rhinolophus affinis*	QMQ39244
*Rhinolophus*. *shameli*	UBB59645
*Rhinolophus*. *pearsonii*	QKE49996.1
*Rhinolophus*. *sinicus (allele4)*	GQ999936
*Rhinolophus*. *sinicus YN (allele7)*	AGZ48803
*Rhinolophus*. *sinicus (allele8)*	GQ999933
*Rhinolophus*. *macrotis*	ADN93471.1
*Rhinolophus*. *pusillus*	ADN93477.1
*Rhinolophus*. *ferrumequinum*	XP_032963186.1
*Hipposideros pratti*	QKE49995.1
*Hipposideros armiger*	XP_019522954.1
*Cynopterus sphinx*	QJF77831.1
*Pteropus vampyrus*	XP_011361275.1
*Rousettus leschenaultii*	BAF50705.1
*Taphozous melanopogon*	QJF77841.1
*Taphozous theobaldi*	QJF77840.1
*Kerivoula pellucida*	QJF77795.1
*Scotophilus kuhlii*	QJF77810.1
*Pipistrellus abramus*	ACT66266
*Tylonycteris robustula*	QJF77813.1

### Effect of RaACE2 polymorphism on entry of SARS-CoV-2 and RaTG13

**Fig 2:**
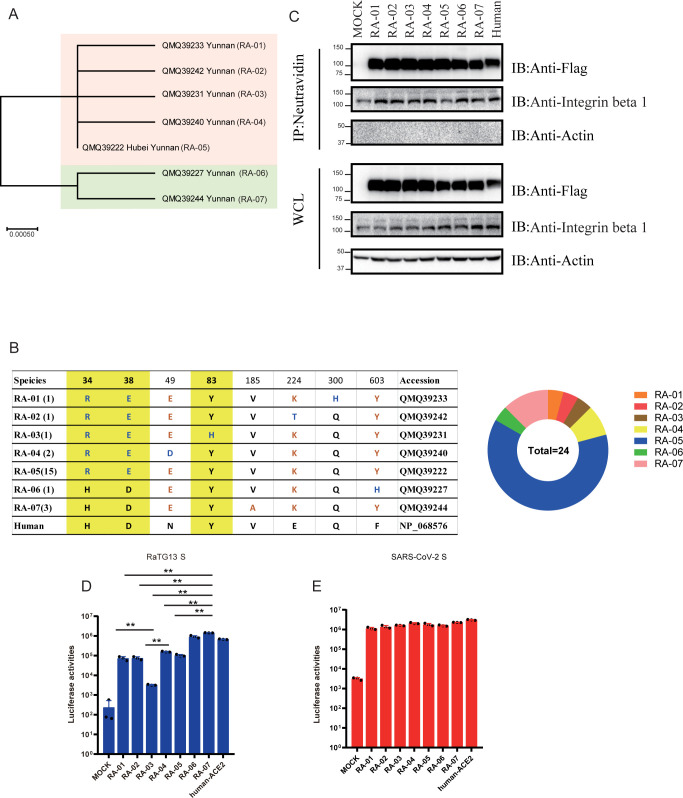
RaACE2 polymorphism confers different susceptibility to RaTG13. (A) Phylogenetic tree of RaACE2 variants. The maximum-likelihood tree (left panel) was produced using MEGA X software, based on the alignment of ACE2 amino acid sequences of *R*. *affinis*. The branch lengths are scaled according to the number of amino acid substitutions per site, indicated by the scale bar. (B) The table of non-synonymous mutations present in the RaACE2 variants. The three of eight key residues which are involved in interacting with the RaTG13 and SARS-CoV-2 spike are shaded with a yellow background. The corresponding eight residues of human ACE2 are indicated at the bottom. (C) Analysis of RaACE2 proteins on the cell surface by cell surface protein biotinylation assay. HEK293 cells transiently overexpressing different RaACE2 and human ACE2 proteins were labeled with EZ-link Sulfo-NHS-LCLC-biotin on ice, and lysed with RIPA buffer. Biotinylated proteins were enriched with NeutrAvidin beads and detected by western blot using mouse monoclonal anti-FLAG M2 antibody. WCL, whole cell lysate. Entry mediated by RaTG13 S pseudovirions (D) and SARS-CoV-2 S pseudovirions (E) into cells expressing different RaACE2 variants. HEK-293 cells transiently expressing different RaACE2 variants were transduced with RaTG13 S pseudovirions or SARS-CoV-2 S pseudovirions and transduction efficiency was measured 40 hrs post-transduction. Experiments were done in triplicate and repeated at least three times. One representative is shown with error bars indicating SEM. Statistical significance is set as * p<0.05 and ** p<0.01 and calculated by t-test.

There are 23 individual bat RaACE2 protein sequences available in the GenBank database, of which one is from the Hubei province of China and the rest 22 are from the Yunnan province of China, we also obtained one additional RaACE2 sequence identical to RA-07 from a sample collected from Yunnan. Sequence alignment of these 24 RaACE2s reveals that there are seven different variants and they share 99 to 100% in amino acid identity with each other and 80.6 to 81.0% in amino acid identity with hACE2 (Fig 2A). Of 24 RaACE2 sequences, one variant (RA-05) has fifteen identical sequences including the one from Hubei province, RA-07 has three identical sequences, and RA-04 has two identical sequences, the remaining four variants (RA-01, RA-02, RA-03, and RA-06) each have one unique sequence (Fig 2B). The sequences of seven variants differ at eight positions, including 34, 38, 49, 83, 185, 224, 300, and 603, of which three residues, 34, 38, and 83, are located in the S protein-interaction interface (Fig 2B) [13, 17, 23]. Among the eight positions, there are three residues different between hACE2 and RA-06, four residues different between hACE2 and RA-07, five residues different between hACE2 and RA-02/RA-04/RA-05, six residues different between hACE2 and RA-01/RA-03 (Fig 2B). Of note, H34, D38, and Y83 are conserved only in RA-06, RA-07, and hACE2, whereas none of them is present in the RA-03 variant. The residues in positions 34 and 38 seem to be co-varied.

Previously, we showed that RA-07 could function as the entry receptor for both RaTG13 and SARS-CoV-2 [18], and we then asked whether any other RaACE2 variants might also mediate virus entry of RaTG13 and SARS-CoV-2 efficiently. The plasmids encoding different RaACE2 variants were transfected into 293 cells and the levels of their expression were determined by western blot using a mouse monoclonal anti-FLAG M2 antibody (Fig 2C). All RaACE2 variants were expressed very well at levels slightly better than hACE2 control. The levels of RaACE2 variants on the cell surface were also evaluated using a surface biotinylation assay, and all RaACE2 variants were present on the cell surface at a similar level (Fig 2C). Actin and integrin-β 1 were used as controls. Next, individual 293/RaACE2 variant-expressing cells were transduced by RaTG13 S protein and SARS-CoV-2 S protein pseudovirions (Fig 2D and 2E). RaTG13 S pseudovirions transduced both 293/hACE2 and 293/RA-07 efficiently, consistent with our previous report [18]. The RA-06 variant also mediated entry of RaTG13 S pseudovirion very effectively at a level similar to hACE2, consistent with the notion that three conserved residues, H34, D38, and Y83, shared by RA-06, RA-07, and hACE2 might be critical for interaction with RaTG13 S protein. In contrast, the remaining five RaACE2 variants showed a significant reduction in transduction efficiency by RaTG13 S pseudovirions. RA-03 variant gave the lowest efficiency, only about 14-fold over vector control (Fig 2D) and close to 50-fold lower than RA-05. Given that RA-03 and RA-05 only differ by a single amino acid at 83, it indicates that Y83 might be critical for the binding of the RaTG13 S protein. Of note, SARS-CoV-2 S pseudovirions entered all RaACE2 variant cells efficiently.

### Effect of RaACE2 polymorphism on receptor binding and membrane fusion

**Fig 3:**
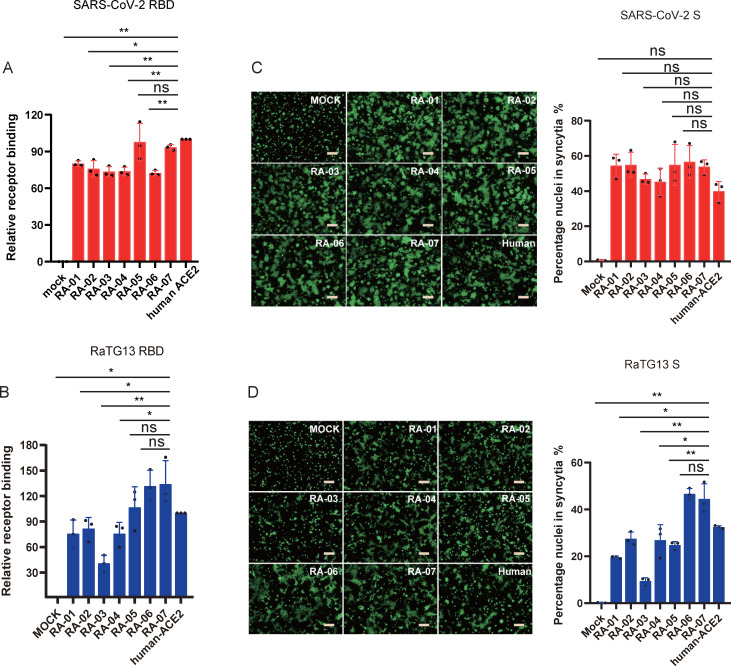
Effect of RaACE2 polymorphism on RBD binding and cell-cell fusion mediated by RaTG13 and SARS-CoV-2 S protein. HEK293 cells transiently expressing RaACE2 variants and human ACE2 were incubated with either SARS-CoV-2 (A) or RaTG13 (B) RBDs, followed by rabbit anti-Strep-tag II antibody incubation and then Alexa Fluor 488-conjugated goat anti-mouse IgG (1:500). Cells were fixed with 1% paraformaldehyde and then analyzed by flow cytometry. The results of the percentage of positive cells from hACE2 binding were set to 100%, the rest was calculated as a percentage of hACE2 binding according to results in flow cytometry analysis. Data are shown as the mean ± SEM. Cell-cell fusion mediated by SARS-CoV-2 (C) and RaTG13 (D) S proteins. HEK293T cells transiently expressing eGFP and S proteins of either SARS-CoV-2 or RaTG13 were detached with trypsin, and overlaid on different ACE2 expressing HEK293 cells. After 4 hrs of incubation, images were taken. The total number of nuclei and the number of nuclei in fused cells for each image were counted. The fusion efficiency was calculated as the number of nuclei in syncytia/total number of nuclei ×100. The scale bar indicates 250 μm. Statistical significance is set as * p<0.05 and ** p<0.01 and calculated by T-test.

Two possibilities could account for the reduced levels of entry of RaTG13 S pseudovirions on RA01 to RA05 variant cells. First, the lower level of transduction might represent a lower affinity of these RaACE2 variants to RaTG13 S protein; second, it might result from inefficient membrane fusion mediated by RaACE2 variants. Receptor binding is the first step of virus entry. Next, we determined the affinity of RaACE2 variants to the RaTG13 receptor binding domain (RBD). As a control, we also included SARS-CoV-2 RBD in our analysis. HEK293 cells transiently expressing different RaACE2 variant proteins were incubated with soluble RaTG13 and SARS-CoV-2 RBDs on ice, and the levels of cells that bound to RBDs were quantified by flow cytometry. SARS-CoV-2 RBD bound RA-05 and RA-07 at a level similar to hACE2 and also showed good binding to the rest of variants at levels of more than 72% of hACE2 (Fig 3A). In contrast, RaTG13 RBD bound to various RaACE2 variants with marked differences. RA-06 and RA-07 variants bound to RaTG13 RBDs with the highest affinity among all variants, even slightly better than hACE2, whereas RA-03 showed the lowest affinity, indicating that residues 34, 38, and 83 of RaACE2 might play critical roles in interaction with RaTG13 S protein. The rest four variants, RA-01, RA-02, RA-04, and RA-05, showed intermediate affinities to RaTG13 RBD (Fig 3B). Overall, the levels of binding of RaTG13 RBD to RaACE2 variants were correlated with their transduction efficiencies by RaTG13 S pseudovirions, indicating that the decrease in transduction efficiency by some RaACE2 variants might result from their low affinities with RaTG13 S protein.

We also evaluated the level of membrane fusion mediated by RaTG13 S proteins on different RaACE2 variants using a cell-cell fusion assay, and SARS-CoV-2 S proteins were also included for comparisons. HEK293 cells transiently expressing hACE2 or RaACE2 variant proteins were incubated with 293T cells co-expressing eGFP with either RaTG13 or SARS-CoV-2 S proteins in the presence of exogenous trypsin. As shown in Fig 3C, extensive syncytia were formed when hACE2-expressing cells were mixed with cells expressing SARS-CoV-2 S proteins, and all RaACE2 variants mediated similar levels of syncytium formation with cells expressing SARS-CoV-2 S proteins. RaTG13 S protein also triggered marked syncytium formation with hACE2 expressing cells, but to a lesser extent compared to SARS-CoV-2 S protein, consistent with our previous report [18]. While RaTG13 S proteins induced slightly higher levels of syncytia on RA-06 and RA-07 variants expressing cells than hACE2 cells, RA-01 and RA-03 gave markedly fewer syncytia than hACE2 (Fig 3D). RA-02, RA-04, and RA-05 showed a similar level of syncytium formation to hACE2 (Fig 3D). Overall levels of cell-cell fusion mediated by RaTG13 S on different RaACE2 variants were similar to their levels of receptor binding, indicating that differences in cell-cell fusion might also result from their variation in affinity with RaTG13 S protein.

### H34 and D38 are critical for interaction with RaTG13 S protein

**Fig 4:**
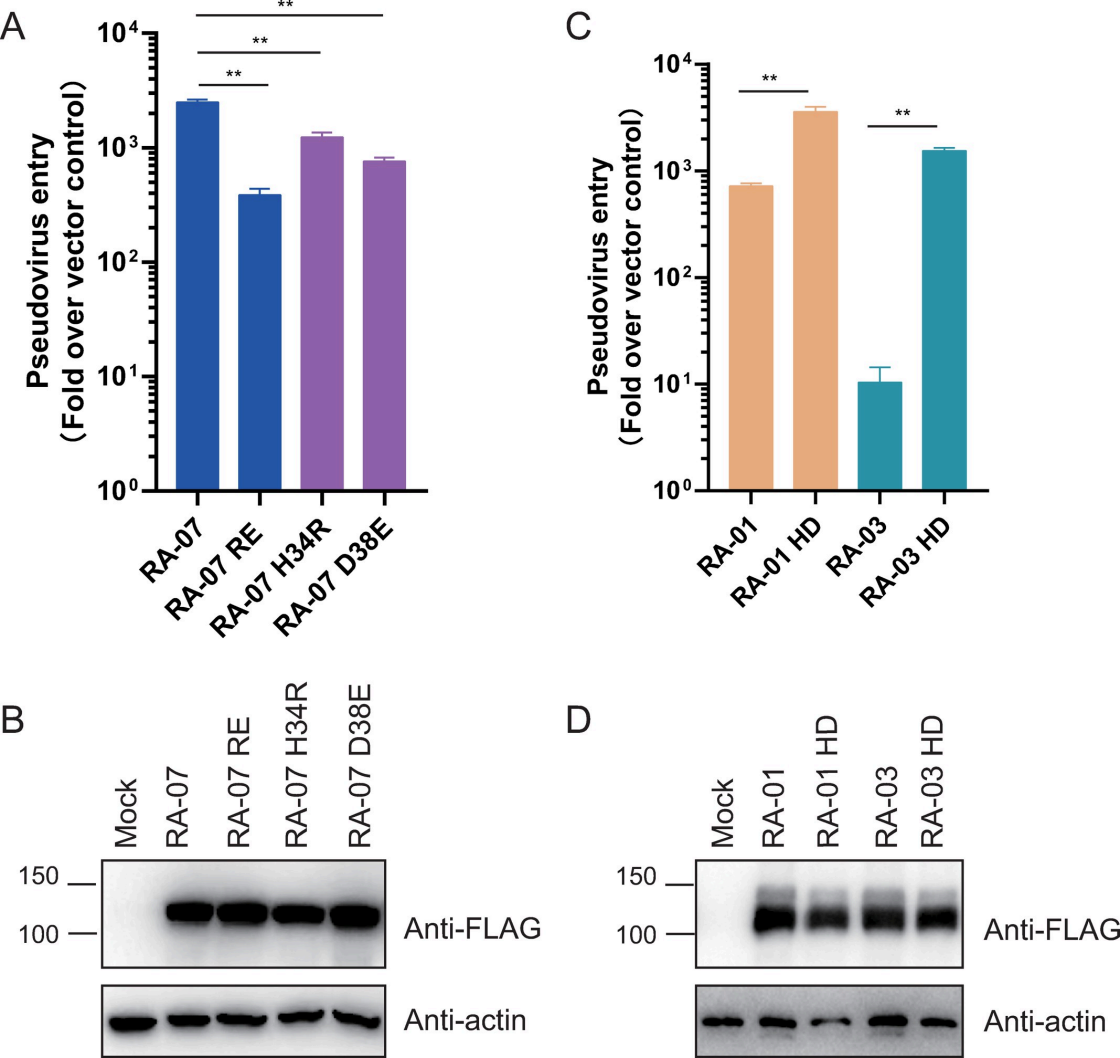
H34 and D38 in RaACE2 are critical for interaction with RaTG13 S protein. Expression of WT and mutant RaACE2 variants, RA-01, RA-03, and mutants (A), RA-07 and mutants (C). HEK293 cells were transfected with plasmids encoding FLAG-tagged RaACE2s using PEI, and lysed at 40 hrs post-transfection. Proteins were detected using an anti-FLAG M2 antibody. The β-actin was used as a loading control. (B) (D) Entry mediated by the S protein of RaTG13 into cells expressing WT and mutant RaACE2 variants, RA-01, RA-03, and mutants (B), and RA-07 and mutants (D). Experiments were done three times, and one representative is shown with error bars indicating SEM. Statistical significance is set as * p<0.05 and ** p<0.01 and calculated by T-test.

Given that H34 and D38 are co-varied and only present in RA-06 and RA-07 variants and both variants showed higher receptor binding and transduction with RaTG13 S protein than other variants, we reasoned that H34 and D38 might act synergistically in interaction with RaTG13 S proteins. Single H34R and D38E mutations in RA-07 decreased RaTG13 S pseudovirion transduction by about 2.0 and 3.3-fold, respectively, whereas H34R/D38E double mutation reduced the infectivity by about 6.4-fold (Fig 4A). None of any mutant RaACE2s showed any marked defect in expression (Fig 4B). We also introduced R34H/E38D double mutation into RA-01 and RA-03 variants (Fig 4C). The double mutation also did not affect RaACE2 expression (Fig 4D). Compared to WT RA-01 and RA-03 variants, R34H/E38D double mutations significantly increased virus entry mediated by RaTG13 S pseudovirions by about 5-fold and 149-fold (Fig 4C), respectively, further confirming the critical roles of H34 and D38 in RaACE2 for RaTG13 virus entry.

### Identification of critical residues in RaTG13 S protein for virus entry

**Fig 5:**
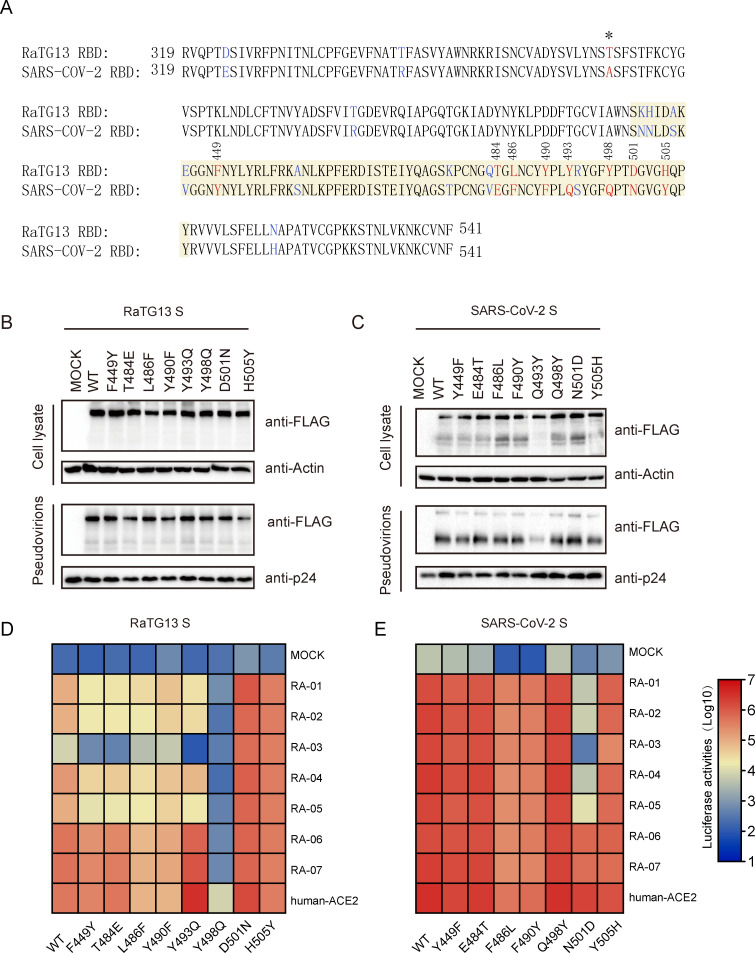
Identification of critical residues in RaTG13 and SARS-CoV-2 S protein for virus entry. (A) Alignment of RaTG13 RBD and SARS-CoV-2 RBD. The RBM of RaTG13 RBD and SARS-CoV-2 RBD are highlighted in yellow background. The mutations chosen are indicated in red. (B) Detection of mutant S proteins of RaTG13 (B) and SARS-CoV-2 (C) in cell lysates and pseudovirions by western blot using anti-FLAG M2 antibody. Top panel, cell lysate; bottom panel, pseudovirions; β-actin and HIV p24 were used as loading controls. Entry mediated by mutant RaTG13 (D) and SARS-CoV-2 (E) S pseudovirions into HEK-293 cells expressing RaACE2 variants. HEK-293 cells transiently expressing RaACE2 variants were transduced with indicated S pseudovirions as well as mutants. Experiments were performed three times, and one representative is shown as the heat map.

Although RaACE2 variants conferred different susceptibility to RaTG13 S pseudovirions (Fig 2D), they showed similar efficiency of transduction by SARS-COV-2 S pseudovirions (Fig 2E). Next, we determined any residues in S protein that might contribute to the disparity. Based on the sequence alignment of receptor binding motifs (RBMs) of RaTG13 (Fig 5A) and SARS-CoV-2 and crystal structures of hACE2/SARS-CoV-2 RBD and hACE2/RaTG13 RBD, we identified residues 449, 484, 486, 490, 493, 498, 501, and 505 as the potential candidates. Single mutations, F449Y, T484E, L486F, Y490F, Y493Q, Y498Q, D501N, and H505Y for RaTG13 S protein and Y449F, E484T, F486L, F490Y, Q493Y, Q498Y, N501D, and Y505H for SARS-CoV-2 S protein, were generated by swapping individual residues at these positions between S proteins of RaTG13 and SARS-CoV-2. All mutant RaTG13 S proteins and all mutant SARS-CoV-2 S proteins except for Q493Y were expressed as well as WT and incorporated into pseudovirion efficiently (Fig 5B and 5C). Because the Q493Y mutant of SARS-CoV-2 S showed a marked defect in S protein incorporation into pseudovirions, it was removed from further analysis. As shown in Fig 5D, Y498Q substitution in RaTG13 S protein showed a significant reduction in transduction on 293/hACE2 cells and almost abolished infection on all RaACE2 variants, consistent with our previous reports that Q might be favored over Y at position 498 of RaTG13 S protein in interaction with various ACE2 [18]. In contrast, single D501N and H505Y mutations in RaTG13 S protein markedly increased infectivity on RA-01 to RA-05 variants, especially on RA-03 by more than 100-fold (Fig 5D), indicating that N and Y might be advantageous on position 501 and 505 in RaTG13 S protein, respectively, and might be responsible for high infectivity of SARS-CoV-2 S pseudovirions on all RaACE2 variants. Of note, while the replacement of Y493 with Q in RaTG13 S showed an increase of infectivity on RA-06, RA-07, and hACE2, it markedly decreased transduction on RA-01, RA-02, RA-04, and RA-05 and completely abolished infectivity on RA-03 (Fig 5D), suggesting that Y might be less favorable than Q in position 493 of RaTG13 S in interaction with the RaACE2 variants with R34 and E38. Similarly, F449Y and T484E single mutations showed minimal effect on RA-06, RA-07, and hACE2, whereas they gave a deeper reduction in transduction on RA-01 to RA-05, especially on RA-03. The L486F and Y490F mutants gave a similar pattern of transduction to WT RaTG13 S protein on all RaACE2 variants but with less efficiency (Fig 5D).

**Fig 6:**
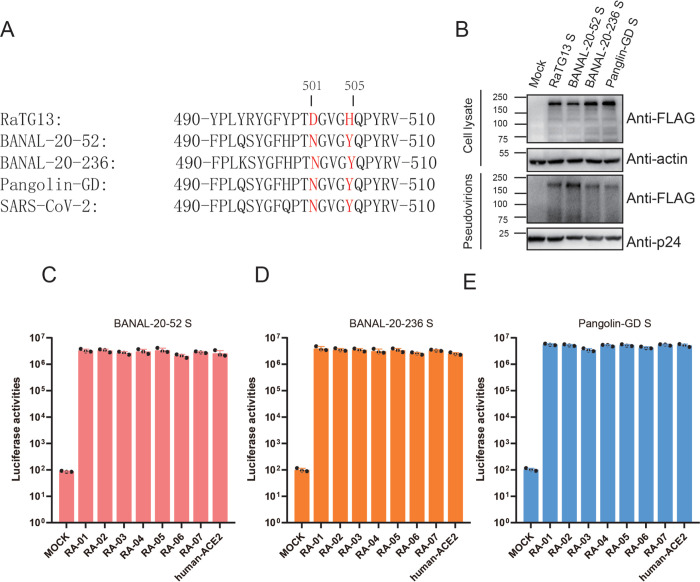
Effect of RaACE2 polymorphism on entry of other SC2r-CoVs. (A) Alignment of amino acid sequences from aa490 to aa510 of RaTG13, BANAL-20-52, BANAL-20-236, Pangolin-GD, and SARS-CoV-2 S proteins. Residues 501 and 505 are labeled in red. (B) Expression and pseudovirion incorporation of RaTG13, BANAL-20-52, BANAL-20-236, Pangolin-GD and SARS-CoV-2 S proteins. Top panel, cell lysate; bottom panel, pseudovirions; β-actin and HIV p24 were used as loading controls. Entry of pseudovirions of BANAL-20-52 (C), BANAL-20-236 (D), Pangolin-GD (E) S proteins on 293/RaACE2s and 293/hACE2 cells.

Among all SARS-CoV-2 S protein mutants, Y449F, E484T, and Q498Y mutants showed minimal or no effect on transduction efficiency on all RaACE2 variants, compared to WT SARS-CoV-2 S protein. F486L, F490Y, and Y505H mutants also showed a similar pattern to WT SARS-CoV-2 S but with about a 2–5 fold reduction in infectivity. Only N501D substitution showed a more than 100-fold decrease in transduction on RA-01 to RA-05, suggesting that N should be favored over D at position 501 of SARS-CoV-2 S protein in interaction with RaACE2 variants. Sequence analysis (Fig 6A) reveals that N501 and H505 are also conserved in S proteins of SARS-CoV-2 related pangolin CoVs [24, 25] and two recently discovered SARS-CoV-2 related bat CoVs (BANAL-20-52 and BANAL-20-236) [26]. We then determined whether their S proteins might use different RaACE2 variants for entry. All three S proteins were expressed well and incorporated into pseudovirions efficiently (Fig 6B), and all three pseudovirions showed similar levels of transduction efficiency on different RaACE2 variants (Fig 6C, 6D and 6E), further confirming the critical role of residue 501 and 505 in interaction with different RaACE2 variants.

**Fig 7:**
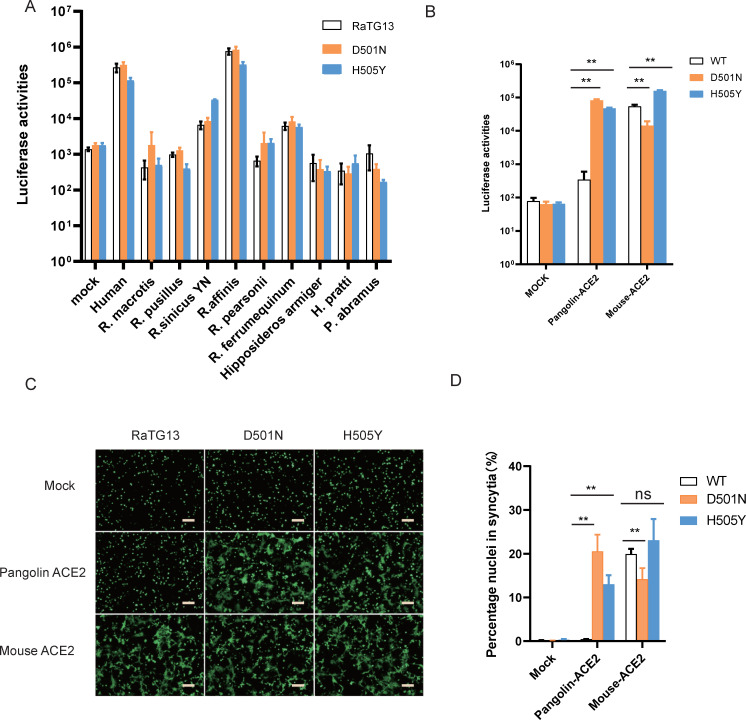
Effect of individual D501N and H505Y changes in RaTG13 S protein on entry of cells expressing bat, pangolin, and mouse ACE2s. Transduction of D501N and H505Y individual mutant RaTG13 S pseudoviruses on cells expressing different bat ACEs (A), pACE2, and mACE2 (B). Effect of D501N and H505Y mutations in RaTG13 S on cell-cell fusion with cells expressing pACE2 and mACE2. The representative images were shown in (C), and the total number of nuclei and the number of nuclei in fused cells for each image were counted. The fusion efficiency was calculated as the number of nuclei in syncytia/total number of nuclei ×100. The scale bar indicates 250 μm. Statistical significance is set as * p<0.05 and ** p<0.01 and calculated by T-test.

Since single D501N and H505Y mutations in RaTG13 S protein showed expanded usage of various RaACE2 variants for virus entry, we then asked whether RaTG13 S with single D501N and H505Y substitutions could increase its susceptibility to other bat ACE2s. Neither D501N nor H505Y mutation showed a marked increase of entry of RaTG13 S pseudovirions on bat ACE2s tested (Fig 7A), indicating that D501N and H505Y alone might not be sufficient for expansion of the host range of RaTG13 among bats.

### Residues 501 and 505 of RaTG13 S are critical for interaction with mouse and pangolin ACE2s

We previously showed that RaTG13 S protein could use mouse but not pangolin ACE2 for virus entry [18]. Since residues in 501 and 505 of RaTG13 S proteins play critical roles in interaction with RaACE2 variants, we then investigated whether these two residues are also important in recognition of pangolin ACE2 (pACE2) and mouse ACE2 (mACE2). Individual D501N and H505Y mutations in RaTG13 S significantly increased virus entry on pACE2 cells by more than 880 and 480-fold (Fig 7B), respectively, compared to mock controls, consistent with the increase of syncytium formation by these two mutants on pACE2 cells (Fig 7C and 7D), indicating that both N501 and Y505 might be important for interaction with pACE2. In contrast, while D501N mutation decreased infectivity on cells expressing mouse ACE2 by 3.8-fold (Fig 7B), H505Y substitution increased transduction by 3-fold (Fig 7B), indicating that mACE2 might favor interactions with D and Y on residues 501 and 505 of RaTG13 S protein over N and H, respectively.

### Effect of T372A mutation in RaTG13 S on entry on bat ACE2s

**Fig 8:**
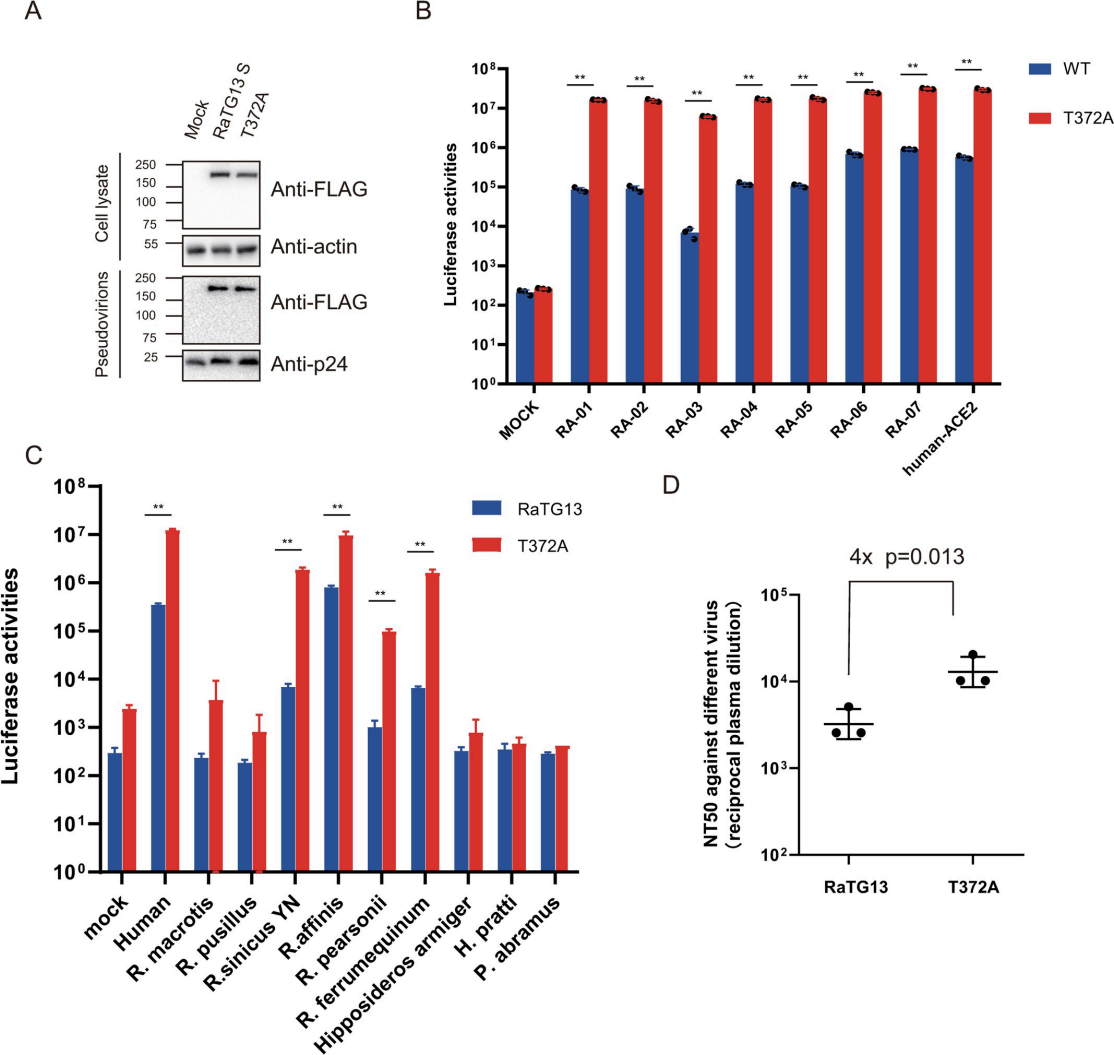
Effect of T372A mutation in RaTG13 S on entry on bat ACE2s and neutralization activities from BANAL-20-52 immunized mouse sera. (A) Western blot analysis of RaTG13 and T372A S proteins in cell lysates and pseudovirions. Top panel, cell lysate; bottom panel, pseudovirions, β-actin, and HIV p24 were used as loading controls. The WT and T372A mutant RaTG13 S pseudovirions were transduced on cells expressing RaACE2 variants (B) and different bat ACE2s (C), and the transduction efficiencies were determined according to luciferase activities. (D) Effect of T372A substitution in RaTG13 on neutralization activities of mouse sera immunized with trimeric BANAL-20-52 S proteins. Mice were immunized with trimeric BANAL-20-52 S proteins twice at days 0 and 14, and sera were collected at day 28 post immunization. The sera from BANAL-20-52 immunized mice were serially diluted and incubated with pseudovirions for 1 hr at 37°C, and the mixture was then incubated with RA-07 expressing cells. After 12 hrs incubation at 37 °C, cells were fed with fresh medium. After another 24 hrs incubation, the transduction efficiencies were measured using Steady-Glo, and neutralization titers were calculated according to the dilution with 50% inhibition. Statistical significance is set as * p<0.05 and ** p<0.01, using an unpaired t-test (Wilcoxon rank test).

Previous reports showed that T372A substitution in RaTG13 S protein might lead to more S1 subunits in “up” or “open” conformation and an increase of receptor binding, resulting in enhancement of the infectivity of RaTG13 S pseudovirions on hACE2 [27]. T372A substitution had no effect on S protein expression and incorporation into pseudovirions (Fig 8A).We then determined whether this mutation had any effect on the entry of different RaACE2 variants and other bat ACE2s or not. Indeed, we observed a close to 52-fold increase in transduction on 293/hACE2 by T372A mutant over WT RaTG13 S pseudovirions (Fig 8B), consistent with the previous report [27]. T372A mutation showed a significant increase in transduction on all RaACE2 variants (Fig 8B). Of note, the RaACE2 variants that gave lower transduction efficiency by WT RaTG13 S showed a higher increase in infectivity by the T372A mutant. Overall transduction efficiency of T372A on the RA-03 variant remained to be the lowest, about 5-fold lower than hACE2, but its fold increase was the highest, about 890-fold over WT RaTG13 S (Fig 8B), indicating that T372A substitution might significantly increase binding affinity with RA-03 over the threshold.

We also selected ACE2s from additional eight bat species known to harbor bat sarbecovirus besides RaACE2 for further evaluation effect of the T372A mutant [28]. Five bat ACE2s unsusceptible to WT RaTG13 S transduction remained unable to be transduced by the T372A mutant. However, *R*. *sinicus* YN, *R*. *personii*, and *R*. *ferrumequinum* ACE2s showed a significant increase in infectivity by T372A mutant by over 270-fold, 96-fold, and 240-fold (Fig 8C), respectively, indicating that T372A mutation in RaTG13 might not only raise the ratio of “up” conformation but also increase the affinity with ACE2s of *R*. *sinicus* YN, *R*. *personii*, and *R*. *ferrumequinum* bats.

Given that the T372A mutant presented clear advantages over WT RaTG13 regarding virus entry on multiple RaACE2 variants and several bat ACE2s (Fig 8B) and WT SARS-CoV-2 A372 viruses also showed higher infectivity than T372 SARS-CoV-2 virus, leading to the obvious question, why does the RaTG13 virus retain T372, not A372, in its S protein? We postulated that extra glycosylation from T372 and RBD in “down” conformation might increase WT RaTG13’s ability to evade potential immunity by steric hindrance that might prevent binding of some neutralizing antibodies, which might result from the previous infection from other SC2r-CoVs, since *R*. *affinis* bats are likely susceptible to infection of multiple different SC2r-CoVs (Fig 6). Sera from mice immunized with trimeric BANAL-20-52 S proteins were collected and their neutralization titers against WT and T372A RaTG13 S pseudoviruses were determined. As shown in Fig 8D, the T372A mutation resulted in an increase in the neutralization activities by 4-fold (p = 0.013), in agreement with our hypothesis.

## Discussion

ACE2 is an important regulator of renin–angiotensin system that regulates blood pressure, fluid, and electrolyte balance, etc, and its polymorphism in humans has been discovered about 20 years ago [29, 30] and has been linked to cardiovascular diseases, hypertension, and diabetic mellitus, etc [31–33]. Given that cardiovascular diseases, hypertension, and diabetic mellitus are important comorbidity factors for COVID-19, it is not surprising that recently the ACE2 polymorphism has also been found to not only be associated with entry efficiency of SARS-CoV-2 [34, 35] but also with COVID-19 outcome [36], indicating that ACE2 polymorphism plays important roles in COVID pathogenesis. Many SARS-CoV related (SC1r-CoV) and SARS-CoV-2 related (SC2r-CoV) bat CoVs including RaTG13 also use human and bat ACE2 as the entry receptor [37–41]. The presence of polymorphism in bat ACE2 has also been reported in several bat species including *R*. *affinis* and *R*. *sinicus* [22, 29]. Analysis of the ratio of nonsynonymous to synonymous mutation (dN/dS) reveals that several critical S-interacting residues in *R*. *sinicus* ACE2s are undergoing positive selection, suggesting potential co-evolution arm race between SC1r-CoV and *R*. *sinicus* ACE2 [22]. In contrast, RaACE2s showed much broader susceptibility to infection of various sarbecoviruses than *R*. *sinicus* ACE2 [38], but there is no obvious positive selection detected among available RaACE2s [22]. In this study, we found that three *R*. *sinicus* ACE2 variants (RS-allele4, 7, 8), accounting for 52% of *R*. *sinicus* population [22], only gave minimal susceptibility to RaTG13 infection (S1 Fig), indicating that *R*. *sinicus* bats might unlikely be the natural host for RaTG13. In contrast, RaACE2 variants show significantly different susceptibility to RaTG13 infection and there are three single residue polymorphisms at positions 34, 38, and 83, critical for entry of RaTG13 virus, whereas the polymorphisms at 34, 38, and 83 seem to have minimal effect on the entry of most of SC1r-CoVs and SC2r-CoVs ([22] and Fig 6), suggesting that RaTG13 virus might not have been well adapted in *R*. *affinis* bats. Of note, among the 18 bat species tested, *R*. *affinis* bat appears to be the only bat species highly susceptible to RaTG13 infection (Fig 1C), whether infection by RaTG13 has any effect on polymorphism of RaACE2 warrants further investigation.

**Fig 9:**
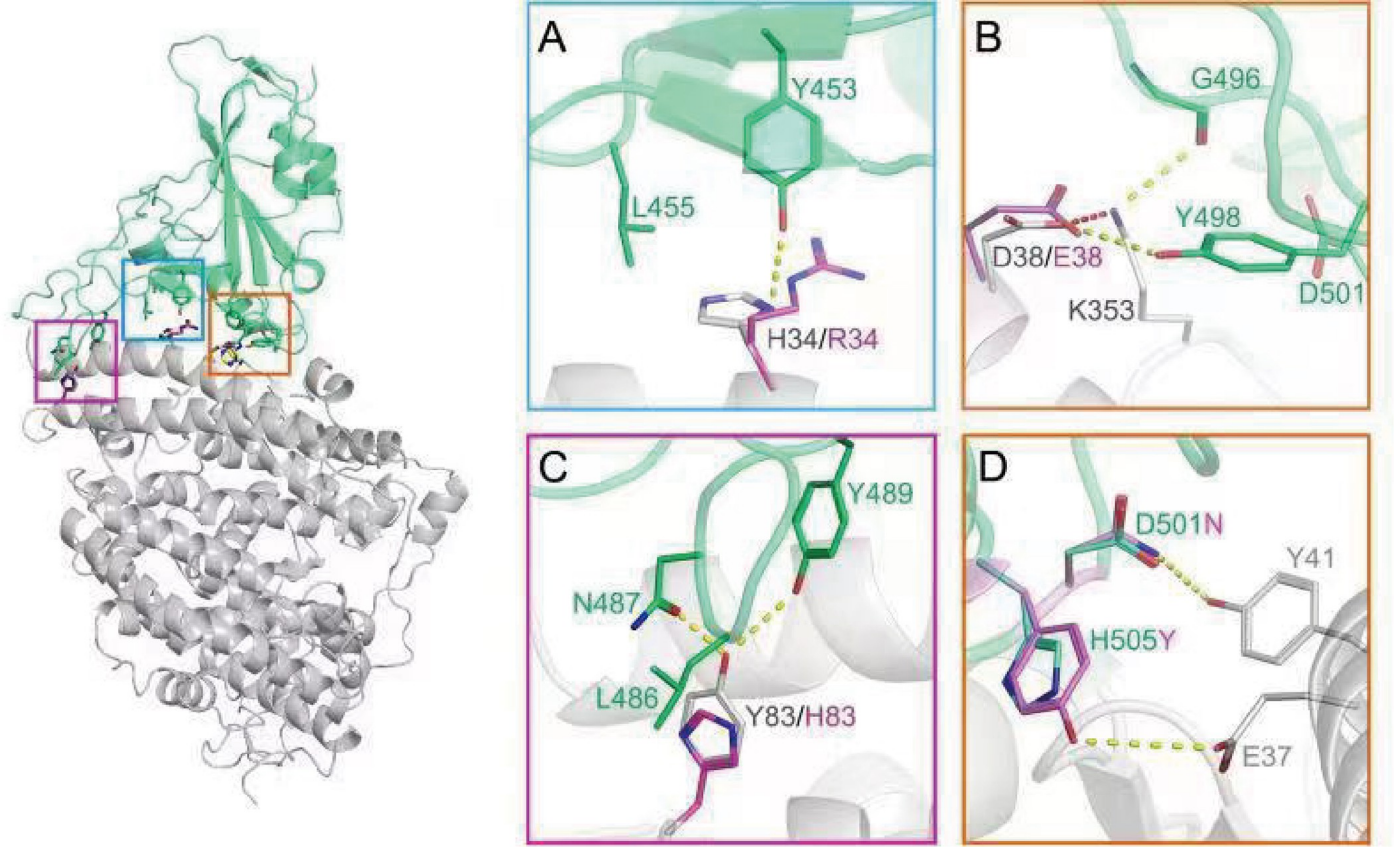
In silico analysis of RaTG13 RBD (green) binding to RA-06/RA-07 (gray) showing key interactions on the binding interfaces. Key interactions with H34/R34, D38/E38, and Y83/H83 are shown in the blue square (A), the orange square (B), and the purple square (C), respectively. Hydrogen bonding and salt bridge are displayed in yellow and red dash lines, respectively. The side chains of H34, D38, and Y83 are labeled in grey, and the side chains of R34, E38, and H83 are labeled in purple. (D) Key interactions with residues 501 and 505 in S protein. D501 and H505 are in green, and N501 and Y505 are in purple.

The residues in position 34 and 38 of RaACE2 seems to be co-varied, and most of the variants possess R34/E38, not H34/D38, in their sequences. However, R34/E38 seems to be somehow detrimental to the infection of RaTG13, and its entry efficiency by RaTG13 pseudovirions was reduced by more than 5-fold, compared to H34/D38 (Fig 4). In silico analysis of RaTG13 binding to RaACE2 (Fig 9) reveals that H34 might form a hydrogen bond with Y453 and van der Waals (vdW) and hydrophobic interactions with L455 in S protein, whereas R34 change might reduce the interaction with Y453 and L455 due to its more flexible side chain (Fig 9A). D38 might form a hydrogen bonding with Y498 as well as vdW interactions with F449 and Y498 in ACE2 and intramolecular salt bridge with K353 that might keep K353 in a position interacting with the main chain of G496 and with side chains of F449 and Y498 in RaTG13 S protein (Fig 9B). In contrast, the E38 change might disrupt the intermolecular salt bridge with K353, making K353 more flexible and decreasing the vdW interactions between K353 of ACE2 and D501 of S protein (Fig 9B). While Y83 in ACE2 might interact with L486, N487, and Y489 of S protein through vdW and hydrogen bonding (Fig 9C), leading to high infectivity of RaTG13 in RA-06 and RA-07, H83 change might weaken its interaction with N487 and Y489 due to smaller and shorter side chain than Y83 (Fig 9C), resulting in poor receptor activity of RA-03 for RaTG13.

The mouse ACE2 (mACE2), not pangolin ACE2 (pACE2), is susceptible to RaTG13 infection ([18] and Fig 7B). Interestingly, mACE2 has residues Q34, D38, and H353, whereas the pACE2 has S34, E38, and K353. In silico analyses show that H353 of mACE2 might form a salt bridge with D501 (S2 Fig), strengthening the interactions between mACE2 and RaTG13 S. In contrast, in pACE2, H34S change might decrease its interactions with Y453 and L455, and D38E change might also disrupt the intermolecular salt bridge with K353, weakening vdW interactions between K353 of ACE2 and D501 of S protein(Fig 9B), similar to RA-03.

Individual D501N and H505Y substitutions in RaTG13 S significantly increased entry on R34/E38 variants and pACE2. D501N substitution might enable an extra hydrogen bond with Y41 of ACE2 and significantly stabilize the binding, whereas H505Y change might form an extra hydrogen bond with E37 of ACE2 and more favorable vdW and hydrophobic interactions with the main chain and K353 of ACE2 (Fig 9D), resulting in a significant increase in the binding affinity with R34/E38 variants and pACE2 and overcoming the energy barrier required for triggering the efficient conformational change of S protein. The S proteins of BANAL-20-52, BANAL-20-236, and pangolin GD also have N501 and Y505, and all of these S proteins efficiently used all RaACE2 variants for virus entry, further confirming the importance of N501 and Y505 on entry on RaACE2 and pACE2. Of note, N501 is present in delta variant and early major circulated strains, whereas Y501 is dominated in alpha, beta, gamma, and omicron variants and strengthens its interaction with both hACE2 and mACE2 [42,43]. N501Y substitution not only enhances SARS-CoV-2 infection and transmission and also broadens the viral host range including mice [44–46]. While Y505 is present in alpha, beta, gamma, and delta variants, H505 becomes dominant in most omicron variants. In addition, D501N and H505Y substitutions had limited effect on entry on H34/D38 RaACE2 variants and various other bat ACE2s (Fig 7A). Given that residues 501 and 505 in the S protein of might play critical roles in the expansion of the potential host range of CoVs, more attention should be paid to monitoring their sequence changes in bat CoVs to minimize the potential zoonotic transmission in the future.

A372 is present in almost all SARS-CoV-2 S proteins, likely resulting from adaptation in humans because of higher infectivity than T372 in human cells [27, 28], and T372A substitution in RaTG13 S protein also showed significantly higher transduction efficiency than WT RaTG13 by causing more receptor binding domain (RBD) in “open” or “up” conformation. In this study, we found that T372A substitution significantly increases infectivity over WT RaTG13 across all RaACE2 variants, especially for RA-03 by an increase of more than 880-fold (Fig 8B), and also markedly enhances entry on ACE2s of *R*. *sinicus* YN, *R*. *personii*, and *R*. *ferrumeiqunum* bats (Fig 8C), indicating that A372 mutant might have higher infectivity than WT RaTG13 in cell culture. Given a significant advantage in infectivity of A372 mutant over WT, why does the RaTG13 viral genome retain T372, not A372? We hypothesize that immune evasion might be one of the contributing factors and extra glycosylation site from T372 and all “down” conformation might enhance the ability of immune evasion from WT RaTG13 over the A372 mutant virus, since *R*. *affinis* bats are likely susceptible to various SC1r-CoV and SC2r-CoV infection (Fig 6). Indeed, A372 pseudovirions showed 4-fold higher neutralization titer to sera from BANAL-20-52 S immunized mice than WT RaTG13 viruses. Alternatively, considering that only one single RaTG13 genome sequence is available, it might not represent the genomes of major RaTG13 variants in bats.

In summary, we find that RaACE2 polymorphisms show different susceptibilities to RaTG13 but not SARS-CoV-2, BANAL-20-52, BANAL-20-236, or pangolin-GD CoV, residues 34, 38, 83 in ACE2 and residues 501 and 505 in the S proteins are critical for interactions between S protein and ACE2, and immune evasion might contribute to the selective advantage of T372 over A372, despite that A372 has significant higher infectivity than T372.

## Materials and methods

### Ethics statement

BALB/c mice were used in this research. The protocol of this study was approved by the Ethic Review Board of Institute of Pathogen Biology, Chinese Academy of Medical Sciences & Peking Union Medical College (GH20002).The experimental protocol was carried out in accordance with the approved guidelines.

### Constructs and plasmids

Human codon-optimized cDNA encoding SARS-CoV-2 S protein (QHU36824.1), S proteins of SARS-like bat CoV RaTG13 (MN996532.1), BANAL-20-52 (GISAID Accession ID: EPI_ISL_4302644), BANAL-20-236 (GISAID Accession ID: EPI_ISL_4302647), and pangolin CoV (Pangolin-GD, hCoV19/pangolin/Guangdong/1/2019|) (EPI_ISL_410721) S protein lacking C-terminal 19 amino acids (aa) were synthesized and cloned into pCMV14-3×Flag (Sigma, St Louis, USA) between the *Hind* III and *Xba* I sites. The lentiviral packaging plasmid psPAX2 was obtained from Addgene (Cambridge, USA). The pLenti-GFP lentiviral reporter plasmid that contains encoding sequences of GFP and firefly luciferase was generously gifted by Fang Li (Duke University). The cDNAs encoding RaACE2, other bat ACE2 orthologs (Table 1) and hACE2 were synthesized by Sango Biotech (Shanghai, China) and cloned into the pCMV14-3×Flag vector between the *Hind* III and *BamH* I sites. The mutations were introduced using a site-directed mutagenesis kit (NEB, Ipswich, MA, USA). All the constructs were verified by sequencing, and the mutant fragments were re-subcloned into the corresponding vectors after the entire coding sequences were verified by sequencing.

### Cell lines

Human embryonic kidney cell lines 293 (#CRL-1573) and 293T expressing the SV40 T-antigen (#CRL-3216) were obtained from American Type Culture Collection (ATCC, Manassas, USA). HEK293 cells stably over expressing recombinant human ACE2 (293/ hACE2) or RaACE2 (293/RaACE2) were established in the lab and maintained in Dulbecco’s modified Eagle medium (DMEM, Gibco, Grand Island, USA) with 10% fetal bovine serum (FBS, Gibco) and 100 units of penicillin, 100 μg of streptomycin, and 0.25 μg of fungizone (PSF, Gibco) per milliliter.

### Antibodies

Mouse monoclonal anti-FLAG M2 antibody and mouse monoclonal anti-β-actin antibody were purchased from Sigma-Aldrich (St Louis, USA). Rabbit polyclonal antibodies against HIV-1 Gag-p24 was purchased from Sino Biological Inc. (Beijing, China). Rabbit polyclonal antibody against integrinβ-1 was purchased from Proteintech (Wuhan, China). Mouse anti-Strep-tag II antibody was purchased from Bioss Biotechnology, (Beijing, China). Donkey anti-rabbit IgG, goat anti-mouse IgG, and rabbit anti-goat IgG were purchased from Jackson ImmunoResearch (West Grove, USA).

### Protein expression and purification

The RaTG13 RBD, SARS-CoV-2 RBD, and BANAL-20-52 trimeric S 2P ectodomain were expressed in Expi293F cells (Gibco). The human codon-optimized DNA sequences encoding the RaTG13 RBD (residues Arg319- Phe541) and SARS-CoV-2 RBD (residues Arg319-Phe541) were inserted into pcDNA3.1(+) with an N-terminal human IgG light chain signal peptide and a C-terminal twin-strep tag. The human codon optimized DNA sequence encoding BANAL-20-52 S ectodomain (residues Met1- Gln1204) with 2P mutation at residues 982–983 and C-terminal foldon tag followed by twin-strep tag was inserted into pcDNA3.1(+). Cells were maintained in serum-free Super 293 HEK293 Expression Medium (KHTEC, Beijing, China) at 37°C, 120 rpm with 8% CO_2_. About 1 mg of the plasmids were transfected into 2×10^9^ 293F cells when the cell density reached 2×10^6^/ml using the Transfection Reagent (KHTEC, Beijing, China) according to manufacturer instructions. Cell culture supernatants were collected 3 days post-transfection and incubated with Strep-Tactin XT Superflow high-capacity resin beads (IBA Lifesciences) for 2 hrs at 4°C, then the beads were washed by wash buffer (20mM Tris, 200mM NaCl, pH = 8.0) before eluted by wash buffer containing 50 mM biotin (Sigma). The eluents were concentrated using Amicon ultra centrifugal filters of 10kDa MWCO (Millipore, USA) followed by an aliquot and stored at -80°C.

### Soluble RBD binding assay

HEK293 cells were transfected with plasmids encoding RaACE2 vs using polyetherimide (PEI, Sigma). About 40 h post-transfection, cells were washed with PBS before being lifted with PBS containing 1 mM EDTA, then immediately washed twice with PBS containing 2% FBS. About 2×10^5^ cells were incubated with 5 μg of soluble RATG13 RBD or SARS-CoV-2 RBD for 1 hr on ice. After washing three times with PBS containing 2% FBS, cells were incubated with Mouse anti-Strep-tag II antibody (1:200 dilution, Bioss Biotechnology, Beijing, China), followed by incubation with Alexa Fluor 488-conjugated goat anti-mouse IgG (1:500). Cells were fixed with 1% paraformaldehyde (PFA) (Solarbio, Beijing, China) and then analyzed by flow cytometry.

### Pseudovirion production and transduction

For pseudotyped virion production, HEK-293T cells were co-transfected with psPAX2, pLenti-GFP, and plasmids encoding individual S protein of SARS-CoV-2 S, RaTG13 S, BANAL-20-52 S, BANAL-20-236 S, or Pangolin-GD at equal molar ratios using PEI. After 40 hrs of incubation, supernatants containing pseudotyped virion were harvested and centrifuged at 1000×g for 10 min to remove cell debris. For transduction, receptor-expressing cells were seeded into 24-well plates at 30%–40% confluence. The next day, cells were inoculated with 500 μl viral supernatant, followed by spinning-inoculation at 800×g for 30 min. After overnight incubation, cells were fed with fresh medium, and cells were lysed with 120 μl of lysis buffer with 1:1 ratio of medium and Steady-glo (Promega, Madison, USA) at 48 hrs post-inoculation. The luciferase activities were quantified by using a Modulus II microplate reader (Turner Biosystems, Sunnyvale, USA). All experiments were performed in triplicate or quadruplicate and repeated at least twice.

### Cell surface protein biotinylation assay

The cell surface biotinylation assay has been performed as previously described [18]. Briefly, 293 cells transiently expressing FLAG-tagged ACE2s were incubated with PBS containing 2.5 μg/ml EZ-linked Sulfo-NHS-LC-LC-biotin (Thermo-Pierce, Waltham, MA, US) on ice for 30 min after washing with ice-cold PBS. The reaction was then quenched using PBS with 100 mM lysine, and then the cells were lysed with RIPA buffer. To pulldown the biotin labeled cell surface proteins, the lysates were incubated with NeutrAvidin beads (Thermo-Pierce, Waltham, MA, US) overnight at 4°C. After washing 3 times with RIPA buffer, samples were resuspended in 30 μl of loading buffer and boiled for 10 min. The level of ACE2 expression on cell surface was determined by western blot using anti-FLAG M2 antibody (1:2000). The β-actin and integrin β1 were used as loading controls for total cell lysates and cell surface proteins, respectively.

### Cell-cell fusion assay

HEK293T cells transiently overexpressing S protein and eGFP were detached by brief trypsin (0.25%, Gibco) treatment, and overlaid on 70% confluent monolayer of ACE2 expressing cells at a ratio of approximately one S-expressing cell to three receptor-expressing cells. After 4 hrs of incubation, images of syncytia were captured with a Nikon TE2000 epifluorescence microscope running MetaMorph software (Molecular Devices, San Jose, USA). All experiments were performed in triplicate and repeated at least three times. Three images for each sample were selected, and the total number of nuclei and the number of nuclei in fused cells for each image were counted. The fusion efficiency was calculated as the number of nuclei in syncytia/total number of nuclei ×100.

### Detection of S protein by Western blotting

HEK293T cells transfected with plasmids encoding either RaTG13 or SARS-CoV-2, BANAL-20-52, BANAL-20-236, Pangolin-GD S proteins were lysed at 40 hrs post-transfection by cell lysate buffer (20 mM Tris-HCl pH 7.5, 150 mM NaCl, 1 mM EDTA, 0.1% sodium dodecyl sulfate (SDS), 1% NP40, 1×protease inhibitor cocktail (Biomake, Houston, USA). After 30 min of incubation on ice, cell lysate was centrifuged at 12,000×g for 10 min at 4°C to remove debris. To pellet down pseudovirions, supernatants of cells transfected with pseudovirion packaging plasmids were collected and centrifuged at 25,000 rpm for 2 h in a Beckman SW41 rotor at 4°C through a 20% sucrose (VWR, Solon, USA) cushion, and virion pellets were resuspended in 40 μl RIPA buffer. The samples were boiled for 10 min and then separated in 10% SDS-polyacrylamide gel electrophoresis (PAGE) gel (Beijing Biotides Biotechnology, Beijing, China) and transferred to nitrocellulose filter membranes (GVS, Sanford, USA). After blocking with 5% milk, the membranes were blotted with anti-FLAG M2 antibodies (1:2,000, Sigma) as primary antibody, followed by horseradish peroxidase (HRP) conjugated secondary antibodies (1:5,000), and visualized with Chemiluminescent Reagent (Bio-Rad, Hercules, USA).

### Pseudovirus neutralization assay

Pseudovirus neutralization assay was performed as previously described with minor modification [16]. RaTG13 WT pseudovirion and RaTG13 T372A pseudovirion were pre-incubated at 37°C for 1 hr with serially diluted mouse anti-BANAL-20-52 S trimer sera that had been incubated at 56°C for 30 min to inactivate complement. The virus-sera mixture was then added onto 293/RaACE2 cells in 96-well plate. The inoculum was replaced with fresh medium after 6 hrs incubation. After 40 hrs incubation, cells were lysed by Steady-glo supplemented with 50% medium and pseudovirion transduction efficiency was measured using Modulus II microplate reader. Experiments were performed in triplicates and repeated at least twice.

### Mice immunization

Polyclonal antibody against BANAL-20-52 trimeric S was produced in 5-week-old female BALB/c mice. Each mouse was immunized with 5 μg BANAL-20-52 trimeric S protein mixed with 0,5 mg/ml aluminum hydroxide adjuvant. The second immunization was performed two weeks after the first immunization and the mice sera were collected 2 weeks later.

### Molecular modeling

The PDB files of the crystal structures of hACE2 binding to SARS-COV-2 spike RBD (6m0j) and RaTG13 spike RBD (7drv) were downloaded from RCSB PDB website (www.rcsb.org). Homology models of different host ACE2s binding to each RBDs were built with SWISS-MODEL web server (swissmodel.expasy.org) and further refined with HADDOCK server (wenmr.science.uu.nl), using water refinement method. The models were viewed, aligned, and analyzed with PyMol software.

## Supporting information

S1 Fig Entry of RaTG13 S pseudovirions on different *R*. *sinicus* ACE2 variants. HEK-293 cells transiently expressing *R*.*affinis* (RA-07) and three *R*.*sinicus* (RS-allele4, 7, and 8) bat ACE2s were transduced with RaTG13 S pseudovirions and the transduction efficiency was detected 40 hrs later according to luciferase activities. Experiments were done in triplicate and repeated at least twice. One representative is shown with error bars indicating SEM. Statistical significance is set as * p<0.05 and ** p<0.01 and calculated by T-test.
(JPG)

S2 Fig Interaction between mouse ACE2 and RaTG13 RBD. Y41 and H353 in mouse ACE2 are labeled in yellow, and D501 of S protein is in green. The salt bridge is displayed in red dash lines and hydrogen bonding is labeled in yellow dash lines.
(JPG)
