# Supplementary figures and images for: Effect of polymorphism in Rhinolophus affinis ACE2 on entry of SARS-CoV-2 related bat coronaviruses
Source: PLoS Pathog. 2023 Jan 23;19(1):e1011116. doi: 10.1371/journal.ppat.1011116 (PMC9904459; doi:10.1371/journal.ppat.1011116)

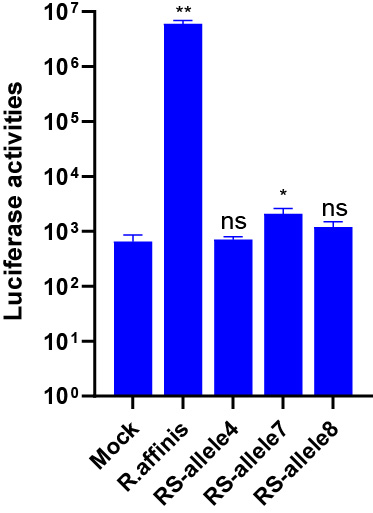

Supplement: S1 Fig — HEK-293 cells transiently expressing R.affinis (RA-07) and three R.sinicus (RS-allele4, 7, and 8) bat ACE2s were transduced with RaTG13 S pseudovirions and the transduction efficiency was detected 40 hrs later according to luciferase activities. Experiments were done in triplicate and repeated at least twice. One representative is shown with error bars indicating SEM. Statistical significance is set as * p<0.05 and ** p<0.01 and calculated by T-test. (JPG) [file ppat.1011116.s001.jpg]

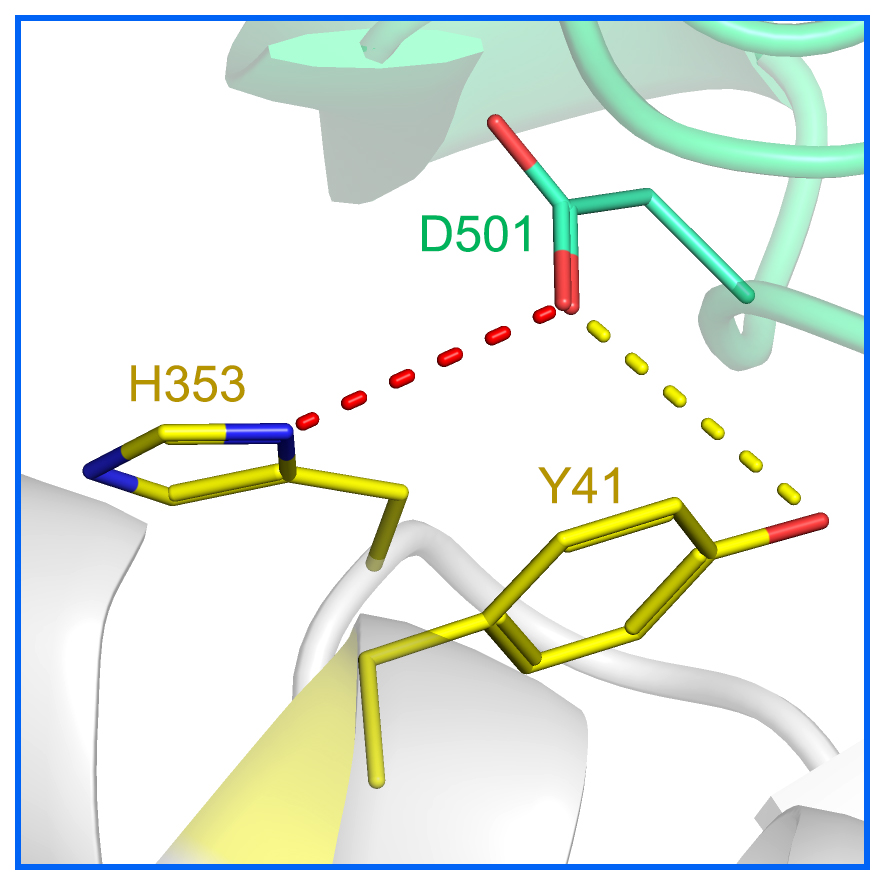

Supplement: S2 Fig — Y41 and H353 in mouse ACE2 are labeled in yellow, and D501 of S protein is in green. The salt bridge is displayed in red dash lines and hydrogen bonding is labeled in yellow dash lines. (JPG) [file ppat.1011116.s002.jpg]
